# 2-Deoxyglucose alleviates migraine-related behaviors by modulating microglial inflammatory factors in experimental model of migraine

**DOI:** 10.3389/fneur.2023.1115318

**Published:** 2023-04-06

**Authors:** Tao Qiu, Yanjie Zhou, Luyu Hu, Zhengming Shan, Yu Zhang, Yuting Fang, Wanbin Huang, Lily Zhang, Shanghua Fan, Zheman Xiao

**Affiliations:** Department of Neurology, Renmin Hospital of Wuhan University, Wuhan, Hubei Province, China

**Keywords:** migraine, glycolysis, inflammation, 2-Deoxy-D-glucose, energy metabolism

## Abstract

**Background:**

Targeting metabolic pathways has emerged as a new migraine treatment strategy as researchers realize the critical role metabolism plays in migraine. Activated inflammatory cells undergo metabolic reprogramming and rely on glycolysis to function. The objective of this study was to investigate the glycolysis changes in the experimental model of migraine and the effect of glycolysis inhibitor 2-Deoxy-D-glucose (2-DG) in the pathophysiology of migraine.

**Methods:**

We used a rat model of migraine that triggered migraine attacks by applying inflammatory soup (IS) to the dura and examined changes in glycolysis. 2-DG was used to inhibit glycolysis, and the effects of 2-DG on mechanical ectopic pain, microglial cell activation, calcitonin gene-related peptides (CGRP), c-Fos, and inflammatory factors induced by inflammatory soup were observed. LPS stimulated BV2 cells to establish a model *in vitro* to observe the effects of 2-DG on brain-derived neurotrophic factor (BDNF) after microglia activation.

**Results:**

In the experimental model of migraine, key enzymes involved in glycolysis such as phosphofructokinase platelet (PFKP), hexokinase (HK2), hypoxia inducible factor-1α (HIF-1α), lactate dehydrogenase (LDH) and pyruvate kinase (PKM2) were expressed in the medullary dorsal horn. While the expression of electronic respiratory transport chain complex IV (COXIV) decreased. There were no significant changes in glucose 6-phosphate dehydrogenase (G6PD), a key enzyme in the pentose phosphate pathway. The glycolysis inhibitor 2-DG alleviated migraine-like symptoms in an experimental model of migraine, reduced the release of proinflammatory cytokines caused by microglia activation, and decreased the expression of CGRP and c-Fos. Further experiments *in vitro* demonstrated that glycolysis inhibition can reduce the release of Iba-1/proBDNF/BDNF and inhibit the activation of microglia.

**Conclusion:**

The migraine rat model showed enhanced glycolysis. This study suggests that glycolytic inhibitor 2-DG is an effective strategy for alleviating migraine-like symptoms. Glycolysis inhibition may be a new target for migraine treatment.

**Graphical abstract:**
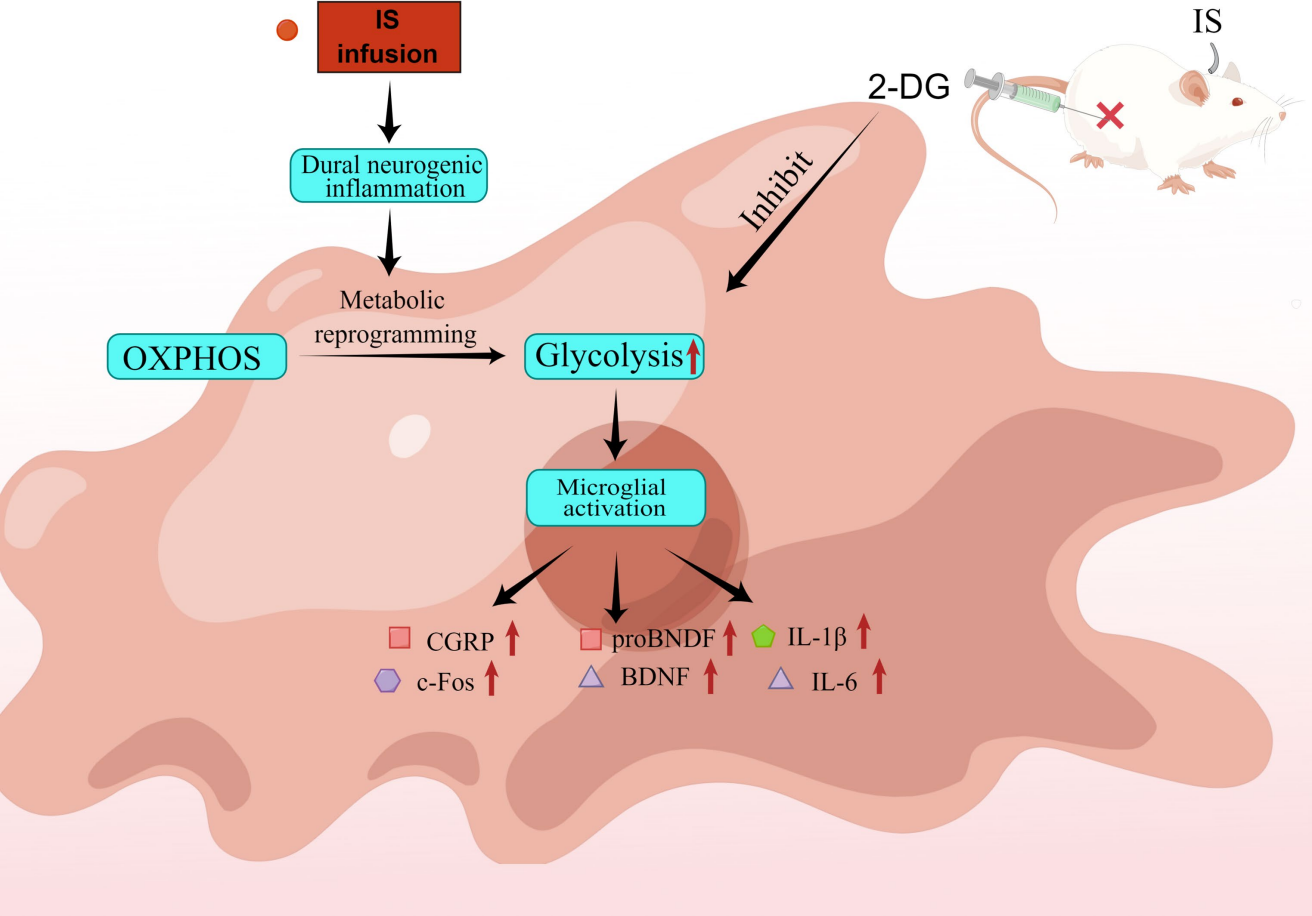
IS dural infusion promotes neurogenic inflammation in rat medullary dorsal horn by enhancing glycolysis. IS dural infusion induced dural neurogenic inflammation in rats. At this time, the energy metabolism of the brain is switched from OXPHOS to glycolysis. Activation of microglia requires the support of glycolysis. The glycolysis inhibitor 2-DG can suppress this process.

## Introduction

Migraine is a neurological disorder associated with cognitive, emotional, and movement disorders, characterized by unilateral pulsatile headache and paroxysmal photophobia, phonophobia, and nausea (1). Out of the 369 diseases and injuries estimated by GBD (Global Burden of Diseases) 2019, migraine was among the most common (2). There is some evidence that aseptic neurogenic inflammation and activation and sensitization of the trigeminal system play an important role in migraine attacks, but the pathophysiology is not fully understood (3).

There are several methods that can be used to induce migraine-like behavior or biomolecular changes in experimental animals. We choose the migraine model induced by IS. The model showed some characteristics related to migraine, such as periorbital and perimasseter mechanical hyperalgesia (4), decreased daily activities (5), head scratching (6), depression and anxiety (7). Migraine animal models show that trigeminal activation results in neuropeptide release, meningeal vasodilation, plasma extravasation, and mast cell degranulation, which are hallmarks of neurogenic inflammation (8). Migraine is closely related to c-Fos and CGRP, markers of trigeminal vascular system activation. As a neural marker closely related to pain, c-Fos is expressed after apparent hyperalgesia in the central nervous system, and due to its immediacy, it can be assumed one of the earliest indicators of successful migraine modeling (9). Increased expression of c-Fos in medullary dorsal horn is believed to be related to central sensitization (10).CGRP is a key factor of migraine as a multifunctional regulatory neuropeptide (11): serum concentrations of CGRP increase during attacks (12) and intravenous infusion of CGRP can cause migraine-like symptoms in migraineurs (13). The most important pro-inflammatory cytokines include IL-1, IL-6, and TNF-α, which may activate injurious neurons. These pro-inflammatory factors are believed to be pain mediators of neurovascular inflammation leading to migraine and play a key role in the occurrence of migraine (14). During migraine attacks, pro-inflammatory factors such as interleukin-6 (IL-6) and interleukin-1β (IL-1β) are elevated in the serum of migraineurs (15–19). These findings suggest that CGRP and other inflammatory factors play an important role in the development of migraine.

BDNF is mainly expressed in the central nervous system, including precursor molecules (proBDNF) and mature forms. BDNF is released by neurons and microglia and plays a key role in neuroinflammation (20). Many studies have shown that activated microglia can release more BDNF, which is involved in many neurological diseases including migraine (21, 22).

As the core process of biological phenomena, metabolism provides energy and building blocks for life activities. In hypoxic environments, glycolysis is the preferred metabolic pathway over oxidative phosphorylation (OXPHOS), and immune cells also undergo this metabolic reprogramming when they are activated (23). Metabolism is an important regulator of inflammation, which broadens the treatment of migraine-related pathology. Although elevated lactate in the cortex of migraine patients has been reported in functional neuroimaging studies (24), no studies on metabolic reprogramming in migraine have been carried out. We, therefore, explored the role of glycolysis in a classic migraine model using the well-known glycolysis inhibitor 2-DG. 2-DG is a glucose analog that is converted by HK into 2-DG-phosphate, which cannot be further metabolized, thereby inhibiting glycolysis.

In this study, 2-DG was applied as a preventive treatment, and we assessed changes in periorbital pain threshold, head scratching, activation of microglia, CGRP, c-Fos, proBDNF, BDNF and inflammatory factors.

## Materials and methods

### Animals

Male adult Sprague–Dawley rats weighing 300–350 g were used. 18 rats were used in the experiment. Under standard laboratory conditions on a 12-h light/dark cycle, rats were housed and maintained. Tap water and rat chow were used *ad libitum*. A week before the experiment, rats were habituated to the experimental environment. The experiments were conducted with the approval of the Committee on Animal Use for Research and Education of the Renmin Hospital of Wuhan University (Wuhan, China). Our evaluation ethics were guided by the guidelines of the International Association for the Study of Pain in Conscious Animals (25). Efforts were made to reduce the number of animals and the degree of suffering.

### Surgical procedure

Rats were anesthetized with isoflurane (3% concentration, 300 ml/min flow). The skin and soft tissues of the rats were sequentially incised to expose the skull. The skull near the sagittal sinus was drilled with a dental drill to expose the dura without penetrating it (26). Then we implanted the guide cannula into the previously drilled cranial window. To minimize trauma to adjacent tissue in an attempt to reduce the chance of infection during surgery. Antibiotics and analgesics were administered post-operatively. After the operation, the state of each rat was observed. And each rat was placed in a single cage to prevent the guide cannula from falling off due to mutual bite. All rats rested for 1 week after surgery.

### Drug administration

Rats were randomly divided into three groups: control group (CON), inflammatory soup model group (IS), and 2-DG pretreatment + IS group (2-DG). The experimental model of migraine was constructed by repeated IS (20 μL, including 2 mM histamine, 2 mM serotonin, 2 mM bradykinin, and 0.2 mM prostaglandin E2 in PBS) dural infusion at the same time each day for at least 7 days. In the control group, rats were injected with the same dose of PBS. In the 2-DG group, 2-DG (500 mg/kg; Meryer) was intraperitoneally injected 30 min before IS infusion.

### Measurement of periorbital pain threshold and head scratching actions

The rat was placed in a plastic tube restraint device, and after the rat was quiet (about 30 min), the periorbital pain threshold of the rat was measured with a von Frey wire and the results were recorded. Baseline pain thresholds were measured before the first IS infusion and after dural infusion. The standard range of Von Frey filaments is 0.008 to 100 g. Von Frey filaments were placed on the periorbital skin of rats. Each test lasted 2 s, with five consecutive tests. Each with a 5 s interval, and a waiting period of at least 30 s between different stimulation intensities (27). The pain threshold is the minimum force that causes the rat to rapidly withdraw its head at least three times out of five stimuli.

The head scratching times of the rats were recorded within 1 h after the IS was injected *via* the tube. Scratching the periorbital and top of the head with the front paws was recorded as the effective number of times.

### Tissue preparation

Two hours after the last administration, the rats were sacrificed after deep anesthesia with sodium pentobarbital. For immunoflurescence, we first exposed rat hearts, flushed systemic blood with PBS through the left ventricle, and then perfused them with paraformaldehyde until they became rigid. Finally, the brain tissue and trigeminal ganglion were removed and fixed in 4% paraformaldehyde solution overnight. The brain tissue and trigeminal ganglion were embedded in paraffin and sliced. For western bloting (WB), the head was placed on ice to remove the brain tissue, and the medullary dorsal horn tissue was separated. The tissue was frozen in liquid nitrogen and stored in −80°C refrigerator.

### Cell culture

BV-2 murine microglia were cultured as described in previous studies (*n* = 3/group) (28). BV-2 cells were cultured in Dulbecco’s modified Eagle’s medium (DMEM) containing 1% 100 U/ml penicillin/streptomycin and 10% fetal bovine serum at 37°C. LPS was diluted in the medium so that the final concentration was 1,000 ng/ml. In six-well plates, 2 ml medium was added to each well of LPS administration group. After 12 h of LPS activation of BV-2 cells with or without 2-DG (1 mM, 2 mM, 4 mM), the medium was harvested and checked for glucose by a glucometer and pH by a pH indicator. CGRP, IL-1β, IL-6, Iba-1, pro-BDNF, BDNF were detected by Western blotting.

### Quantitative real-time polymerase chain reaction

The total RNA was extracted from tissues using TRIzol reagent according to manufacturer’s instructions. Following the manufacturer’s instructions, 1 μg of total RNA was used to synthesize cDNA using the SweScript All-in-One First-Strand cDNA Synthesis SuperMix Kit (servicebio). The Lightcycler 4800II instrument was used for quantitative RT-PCR of HK2, PKM2, PFKP, LDH and HIF-1α genes. TaqMan primers were purchased from Sangon Biotech. Gene expression was measured by comparative CP. Standardize the expression level in the test sample as an endogenous reference β-actin level. The primer sequence of HK2 was: forward, 5′-TGATCGCCTGCTTATTCACGG-3′ and reverse, 5′-AACCGCCTAGAAATCTCCAGA-3′. The primer sequence of PKM2 was: forward, 5′-GCCGCCTGGACATTGACTC-3′ and reverse, 5′-CCATGAGAGAAATTCAGCCGAG-3′. The primer sequence of PFKP was: forward, 5′-CGTCCAGCACCTCCTTT-3′ and reverse, 5′-ACGGACAGCAGCATTCA-3′. The primer sequence of LDH was: forward, 5′-AGGAGAAACACGCCTTGATTTAG-3′ and reverse, 5′-ACGAGCAGAGTCCAGATTACAA-3′. The primer sequence of HIF-1α was: forward, 5′-ACCTTCATCGGAAACTCCAAAG-3′ and reverse, 5′-ACTGTTAGGCTCAGGTGAACT-3′. The primer sequence of β-actin was: forward, 5′-AGGGAAATCGTGCGTGAC-3′ and reverse, 5′-CGCATTGCCGATAGTG-3′. Data are expressed as mean ± SD.

### Western blotting

The medulla dorsal horn was cut into small pieces, and the tissues were washed three times by adding an appropriate amount of cold PBS after weighing. Add 100 μL of lysis buffer and corresponding protease inhibitors per 50 mg of tissue. Protein concentration was measured by using the BCA protein assay kit (servicebio). After heating the mixture with loading buffer at 100°C for 5 min, the collected proteins were loaded and separated on a 10% SDS PAGE gel (Epizyme) and electrically transferred to PVDF membranes. Membranes were incubated with the primary antibody overnight at 4°C following 5% nonfat milk blocking for 2 h at room temperature. The primary antibody was diluted as follows: PFKP (1:1,000; servicebio), HK1 (1:1,000; servicebio), G6PD (1:1,000; servicebio), COXIV (1:1,000; proteintech), CGRP (1:1,000; Santa Cruz), c-Fos (1:1,000; Santa Cruz), Iba-1 (1:1,000; woko), IL-6 (1:1,000; proteintech), IL-1β (1:1,000; proteintech), β-actin (1:2,000; servicebio), pro-BDNF (1:2,000; abcam), BDNF (1:2,000; abcam). β-actin served as respective controls. Incubation with the secondary antibodies followed for 2 h at room temperature. Enhanced chemiluminescence (ECL; biosharp) luminescent solution was used to visualize bands. Images were acquired using a chemiluminescence imaging system (BIO-RAD ChemiDoc Touch). The acquired images were analyzed by using Image J software (Image J 1.48v).

### Immunofluorescence staining

Trigeminal ganglion slices with a thickness of 4 μm were then prepared. Sections were dewaxed in xylene. Then the alcohol grade was lowered for rehydration. Sections were incubated with 0.3% Triton X-100 for 15 min, blocked with 5% BSA for 60 min at room temperature, and then incubated with primary antibody overnight at 4°C. PBS was washed three times after each operation. The primary antibody was diluted as follows: CGRP (1:100; Santa Cruz), Iba-1 (1:500; woko), PFKP (1:200; servicebio), GFAP (1:200; GeneTex), NeuN (1:500; proteintech). After washing with PBS, sections were incubated with mouse secondary antibody (488 nm, green; servicebio). Anti-mouse (488 nm, green; servicebio), anti-rabbit (CY3, red; servicebio) and anti-goat (FITC, green; servicebio) were used. DAPI (servicebio) was incubated for 10 min for staining nuclei. Capture images under the objective of an upright microscope.

### Statistical analysis

GraphPad Prism 8 was used for statistical analysis and graph generation. The variance homogeneity test and the normality test were performed. Unpaired t test was used to assess the difference between the two groups and a one-way analysis of variance (ANOVA) was used to assess the difference among multiple groups. Graphical representation of data is made with bar and line charts. All results are expressed as mean  ±  SD. Differences were considered statistically significant at *p* < 0.05.

## Results

### IS dural infusion increased glycolysis In experimental models of migraine

**Figure 1:**
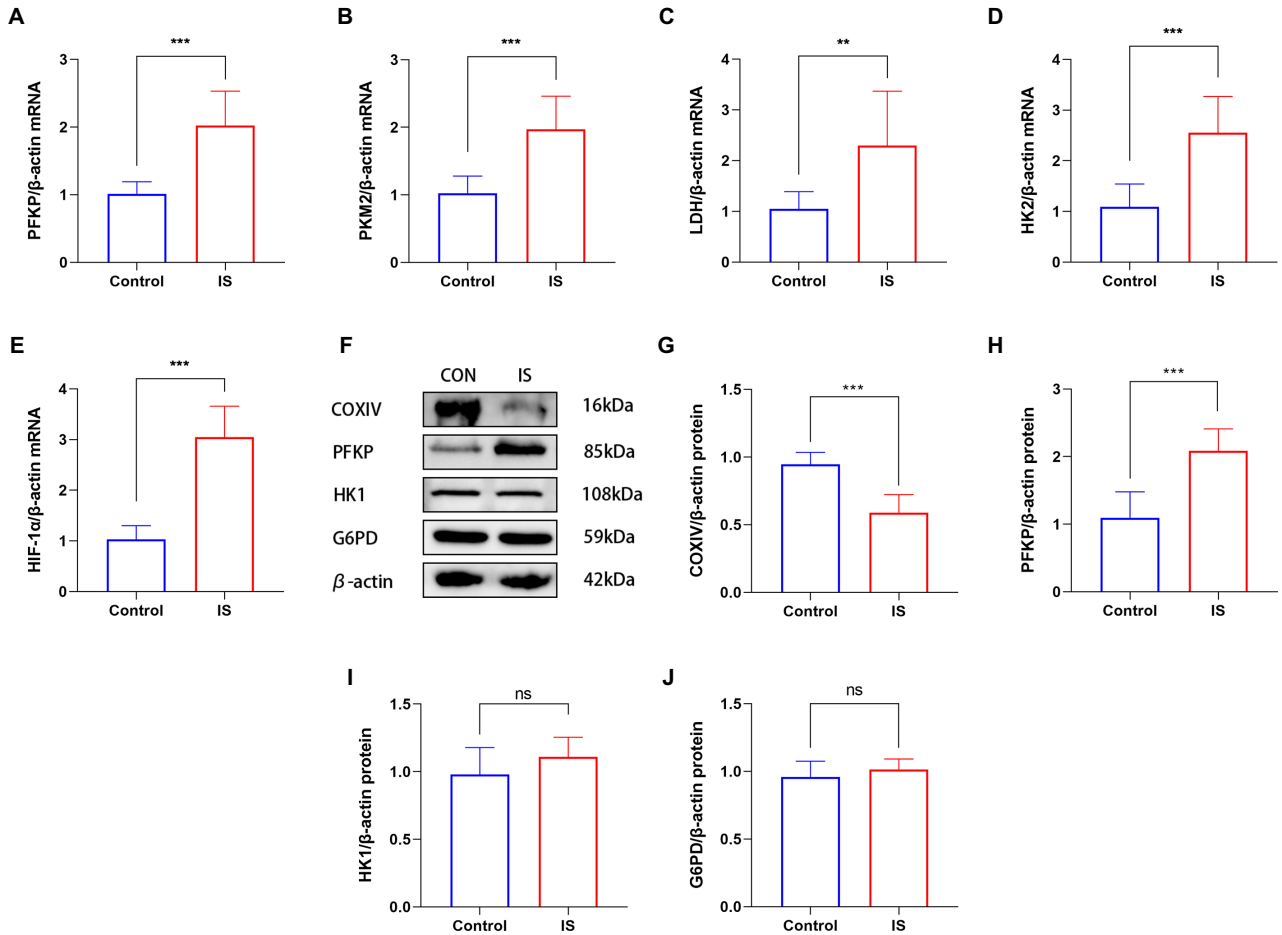
IS dural infusion induced the upregulation of glycolysis in the medulla dorsal horn. **(A–E)** Real time quantitative polymerase chain reaction showed that compared with the control group, the expression of glycolysis related genes (including PFKP, HK2, PKM2, LDH and HIF-1α) in migraine model group increased. Data were expressed as mean ± SD (*n* = 3 per group). ^**^p < 0.01, ^***^p < 0.001. **(F)** WB analysis of PFKP, COXIV, HK1 and G6PD protein levels in the medulla dorsal horn. **(G)** Quantification of the WB showed that the expression of COXIV protein in the medulla dorsal horn of IS group was lower than that of the CON group. Data were expressed as mean ± SD (*n* = 3 per group). ^***^p < 0.001. **(H)** IS dural infusion enhanced PFKP protein levels in the medulla dorsal horn. Data were expressed as mean ± SD (*n* = 3 per group). ^***^p < 0.001. **(I)** Quantification of the WB showed no significant difference in HK1 protein expression in the medulla dorsal horn. Data were expressed as mean ± SD (*n* = 3 per group). **(J)** Quantification of the WB showed no significant difference in G6PD protein expression in the medulla dorsal horn. Data were expressed as mean ± SD (*n* = 3 per group).

To explore whether glycolysis increased during the experimental models of migraine, we detected the gene expression of important enzymes related to glycolysis, such as PFKP, HK2, HIF-1α, LDH and PKM2. Compared with CON group, quantitative real-time polymerase chain reaction showed that PFKP (2.02 ± 0.51), HK2 (2.55 ± 0.72), HIF-1α (3.05 ± 0.61) and PKM2 (1.97 ± 0.49) mRNA expression increased in migraine model group (Figures 1A–E). In addition, we examined the key enzymes of the glycolysis and pentose phosphate pathways, and COXIV protein levels in medullary dorsal horn (Figure 1F). The expression of COXIV (0.59 ± 0.13) in the experimental model of migraine was significantly reduced (Figure 1G). In the medullary dorsal horn of the experimental migraine model, the key glycolysis enzyme PFKP (2.09 ± 0.32) was significantly higher than that in the control group (1.10 ± 0.38; Figure 1H). However, another key glycolysis enzyme, HK1 (1.11 ± 0.14), did not change significantly (Figure 1I). Moreover, G6PD (1.01 ± 0.08), a key enzyme in the pentose phosphate pathway, was almost unchanged (Figure 1J). These results indicated that oxidative phosphorylation is impaired but glycolysis is enhanced in migraine animal models.

### Pretreatment with 2-DG alleviated IS dural infusion induced periorbital and facial hyperalgesia

**Figure 2:**
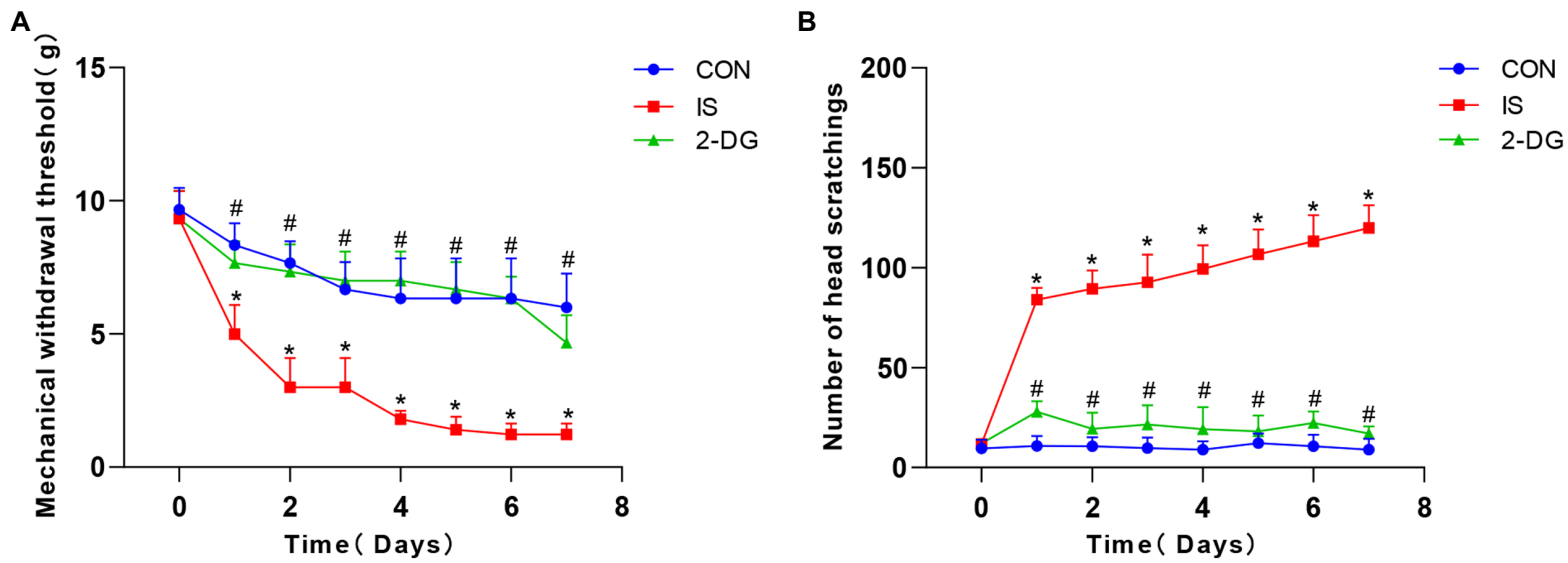
2-DG alleviated nociceptive behavior induced by repeated IS dural infusion. **(A)** The pain threshold of IS group decreased with the days of administration and was lower than that of the CON group, and 2-DG alleviated the IS-induced decrease in pain threshold. Data were expressed as mean ± SD (*n* = 6 per group). ^*^represent IS versus control; #represent 2-DG versus IS. ^*^p < 0.05, # *p* < 0.05. **(B)** The number of head-scratching in IS group was significantly higher than that of the CON group, and 2-DG alleviated the IS-induced increase in head-scratching frequency. Data were expressed as mean ± SD (*n* = 6 per group). ^*^represent IS versus control; #represent 2-DG versus IS. ^*^p < 0.05, # *p* < 0.05.

Daily intraperitoneal injection of 2-DG 0.5 h before IS dural infusion for 7 days significantly inhibited the development of facial friction (Figure 2A) and tactile allodynia (Figure 2B). In rodents, stimulatory factors in the trigeminal region produce characteristic directional grooming behaviors. We used this migraine-like feature as a tool for assessing distress. To investigate the effect of glycolysis inhibitor 2-DG on the rat model of migraine, 2-DG was administered intraperitoneally. The mechanical thresholds of the periorbital region significantly decreased in the IS group (1.23 ± 0.41) compared to the CON group (6.00 ± 1.27). The administration of 2-DG (4.67 ± 1.03) increased the mechanical thresholds of migraine rats. Compared with those of the CON group (9.00 ± 5.33), the number of head scratchings in the IS group (119.83 ± 11.37) significantly increased. The administration of 2-DG (17.00 ± 3.52) decreased the head scratching times of migraine rats. These results indicated that glycolysis may participates in migraine-associated allodynia.

### 2-DG treatment reduced IS-induced astrocyte proliferation, but had no significant effect on neurons

**Figure 3:**
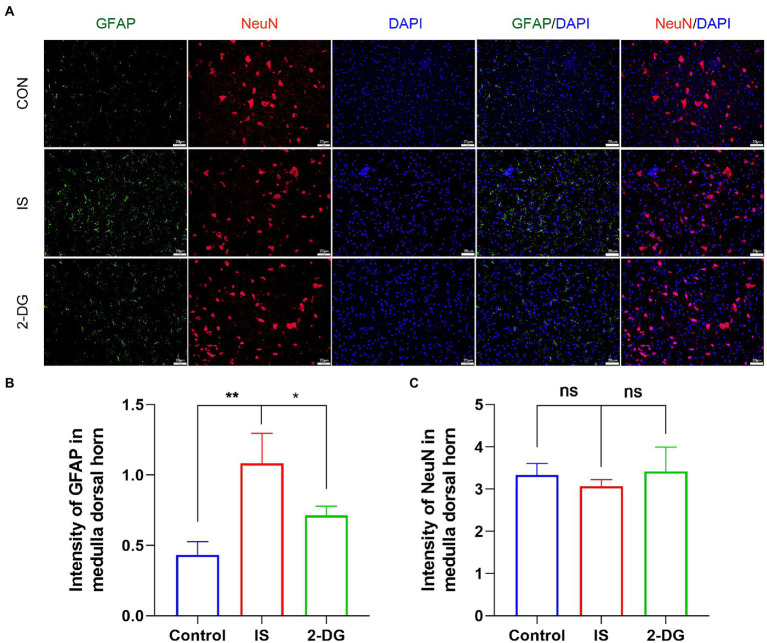
2-DG inhibited IS-induced astrocyte proliferation, but had no significant effect on neurons. **(A)** The immunoreactivity of GFAP and NeuN in medullary dorsal horn. **(B)** Quantification of GFAP immunofluorescence intensity in medulla dorsal horn. Data were expressed as mean ± SD (*n* = 3 per group). ^*^p < 0.05, ^**^p < 0.01. **(C)** Quantification of NeuN immunofluorescence intensity in medulla dorsal horn. Data were expressed as mean ± SD (*n* = 3 per group).

We further explored the effects of 2-DG treatment on astrocyte proliferation and neuron loss (Figure 3A). We found that the astrocytes proliferated in the medullary dorsal horn after IS stimulation in rats (1.08 ± 0.21). The treatment of 2-DG (0.71 ± 0.06) can inhibit the proliferation of astrocytes (Figure 3B). However, no significant neuronal loss was observed in the medullary dorsal horn after IS stimulation (3.07 ± 0.16). No significant difference was observed between the rats treated with 2-DG (3.42 ± 0.57) and the control group (3.33 ± 0.28; Figure 3C).

### IS dural infusion induced higher c-Fos and CGRP expression in the medullary dorsal horn, which was alleviated by pretreatment of 2-DG

**Figure 4:**
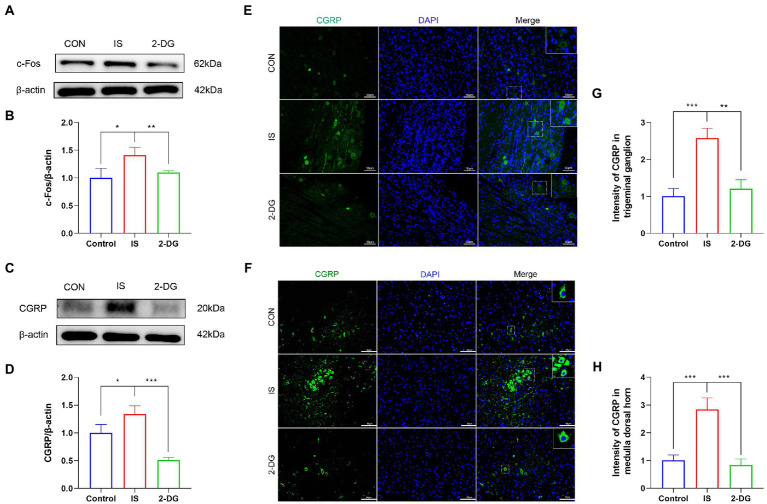
2-DG inhibited IS-induced upregulation of c-Fos and CGRP protein. **(A)** WB of c-Fos protein in the medullary dorsal horn. **(B)** Infusion of IS increased c-Fos protein levels in the medulla dorsal horn, and the expression of c-Fos was significantly decreased in the 2-DG group compared with the IS group. Data were expressed as mean ± SD (*n* = 3 per group). ^*^p < 0.05, ^**^p < 0.01. **(C)** WB of CGRP protein in the medullary dorsal horn. **(D)** Infusion of IS increased CGRP protein levels in the medulla dorsal horn, and the expression of CGRP was significantly decreased in the 2-DG group compared with the IS group. Data were expressed as mean ± SD (*n* = 3 per group). ^*^p < 0.05, ^***^p < 0.001. **(E)** The immunoreactivity of CGRP in the trigeminal ganglion. **(F)** The immunoreactivity of CGRP in the medullary dorsal horn. **(G)** Quantification of CGRP immunofluorescence intensity in trigeminal ganglion. Data were expressed as mean ± SD (*n* = 3 per group). ^**^p < 0.01, ^***^p < 0.001. **(H)** Quantification of CGRP immunofluorescence intensity in medulla dorsal horn. Data were expressed as mean ± SD (*n* = 3 per group). ^***^p < 0.001.

To investigate the contribution of glycolysis to the experimental models of migraine, we measured the expression of c-Fos in the medullary dorsal horn by WB (Figure 4A). Compared with the control group, the IS group (1.41 ± 0.14) showed higher c-Fos protein levels. However, intraperitoneal administration of 2-DG (1.10 ± 0.03) reduced the level of c-Fos protein expression (Figure 4B).

To investigate the effect of inhibition of glycolysis on the alleviation of migraine, we measured the expression of CGRP in the medulla dorsal horn by western blotting (Figure 4C). The protein levels of CGRP were elevated in the IS group (1.34 ± 0.15) compared with the CON group (1.00 ± 0.15), whereas 2-DG (0.51 ± 0.04) treatment decreased CGRP levels (Figure 4D). Immunofluorescence staining were also conducted to detect the expression of CGRP in trigeminal ganglion and medulla dorsal horn. The fluorescence intensity of CGRP in the trigeminal ganglia of the IS group (2.57 ± 0.27) was increased compared with that of the CON group (1.00 ± 0.21). However, 2-DG (1.21 ± 0.24) treatment attenuated the increase in CGRP immunofluorescence intensity induced by inflammatory soup infusion (Figures 4E,G). Similarly, the fluorescence intensity of CGRP in the medulla dorsal horn Figures 4F, H in the IS group (2.83 ± 0.42) was significantly higher than that in the CON group (1.09 ± 0.20), and this increase was suppressed in the 2-DG group (0.82 ± 0.23).

### 2-DG suppressed microglial activation following repeated IS dural stimulation

**Figure 5:**
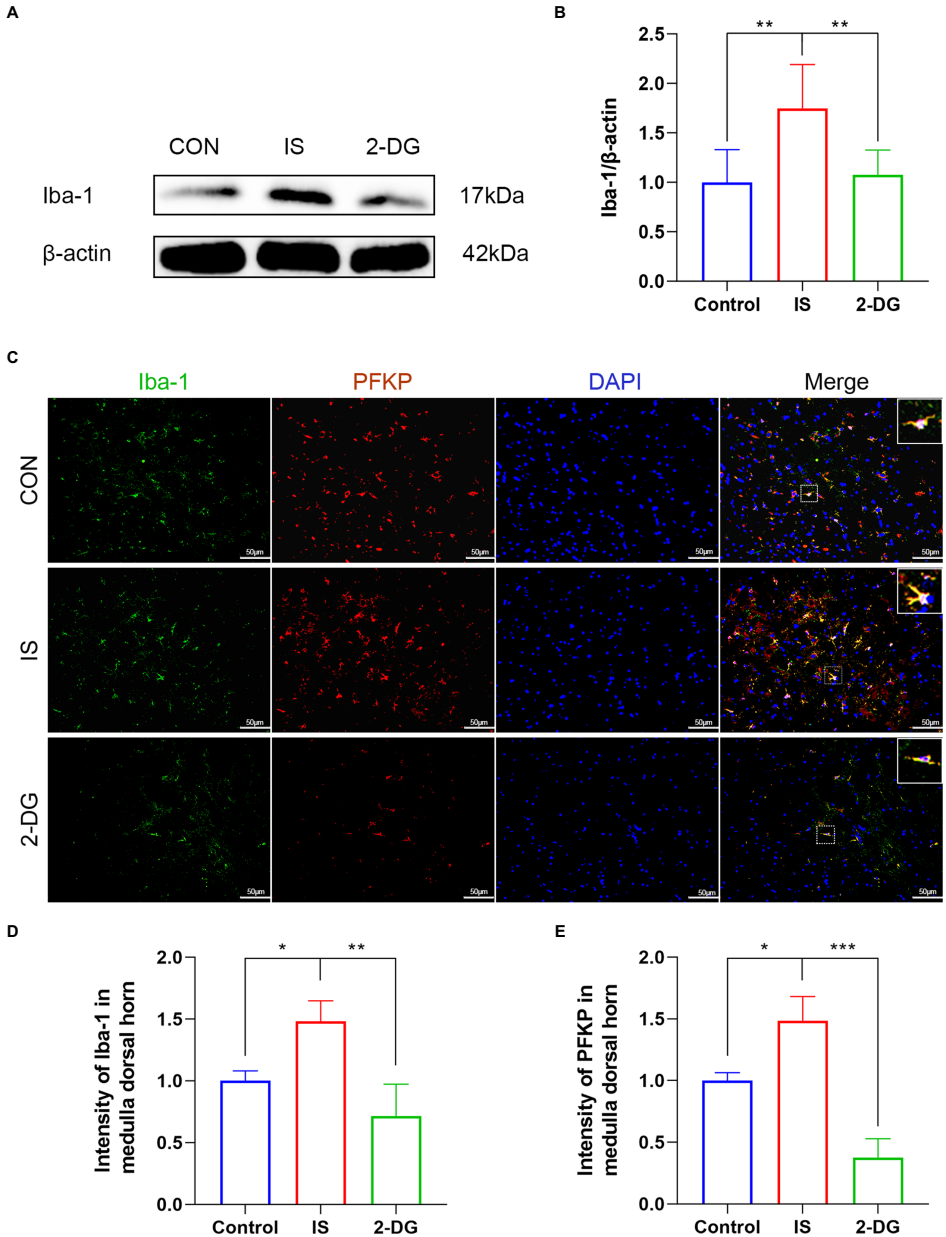
2-DG inhibited IS-induced microglial activation in the medulla dorsal horn. **(A)** WB of Iba-1 protein in the medullary dorsal horn. **(B)** Infusion of IS increased Iba-1 protein levels in the medulla dorsal horn, and the expression of Iba-1 was significantly decreased in the 2-DG group compared with the IS group. Data were expressed as mean ± SD (*n* = 3 per group). ^**^p < 0.01. **(C)** The immunoreactivity of Iba-1 and PFKP in the medullary dorsal horn. **(D)** Quantification of Iba-1 immunofluorescence intensity in medulla dorsal horn. Data were expressed as mean ± SD (*n* = 3 per group). ^*^p < 0.05, ^**^p < 0.01. **(E)** Quantification of PFKP immunofluorescence intensity in medulla dorsal horn. Data were expressed as mean ± SD (*n* = 3 per group). ^*^p < 0.05, ^***^p < 0.001.

Iba-1 is a measure of microglia reactivity after central nervous system injury. And when microglia are activated, Iba-1 protein expression increases (29). To investigate the effect of inhibiting glycolysis on migraine microglia activation, we used WB to detect the expression of Iba-1 in the medulla dorsal horn (Figure 5A). WB analysis showed that compared with the control group, the level of Iba-1 in the IS group (1.75 ± 0.44) was significantly increased. Furthermore, intraperitoneal injection of 2-DG (1.07 ± 0.24) significantly decreased Iba-1 levels (Figure 5B). This suggests that the inhibition of glycolysis may inhibit the activation of microglia. Likewise, the expression of Iba-1 in the medulla dorsal horn was detected using immunofluorescence. Compared with the CON group, immunofluorescence showed that the expression of Iba-1 in the IS group (1.48 ± 0.16) was significantly increased and could be inhibited by 2-DG (0.71 ± 0.26). In addition, the double-labeled immunofluorescence studies further revealed we found that Iba-1 co-stained with PFKP in the microglia cytoplasm, and the co-expression of both was increased in activated microglia.

### IS dural infusion induced the release of pro-inflammatory factors following microglial activation, which can be inhibited by 2-DG

**Figure 6:**
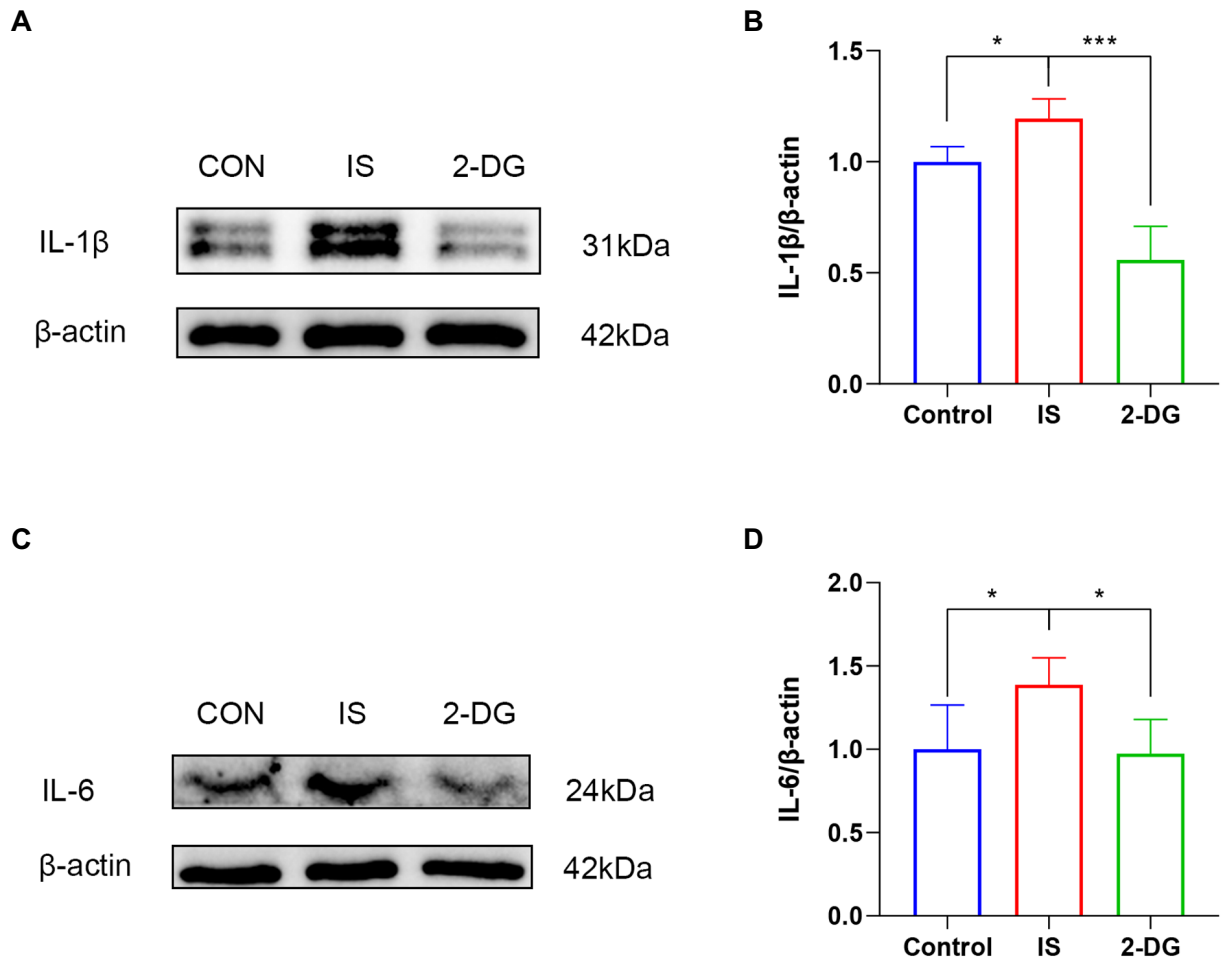
2-DG inhibited IS-induced release of pro-inflammatory factors in the medulla dorsal horn. **(A)** WB of IL-1β protein in the medullary dorsal horn. **(B)** Infusion of IS increased IL-1β protein levels in the medulla dorsal horn, and the expression of IL-1β was significantly decreased in the 2-DG group compared with the IS group. Data were expressed as mean ± SD (*n* = 3 per group). ^***^p < 0.001, ^*^p < 0.05. **(C)** WB of IL-6 protein in the medullary dorsal horn. **(D)** Infusion of IS increased IL-6 protein levels in the medulla dorsal horn, and the expression of IL-6 was significantly decreased in the 2-DG group compared with the IS group. Data were expressed as mean ± SD (*n* = 3 per group). ^*^p < 0.05.

The activation of microglia and subsequent release of various pro-inflammatory cytokines such as IL-6 and IL-1β lead to hyperalgesia or allodynia in experimental migraine models (30, 31). To investigate the effect of inhibiting glycolysis on the release of pro-inflammatory factors in migraine, we used WB to examine the expression of IL-1β and IL-6 in the medulla dorsal horn (Figures 6A,C). WB analysis showed that compared with the control group, the levels of IL-1β (1.20 ± 0.09) and IL-6 (1.38 ± 0.16) in the IS group were significantly increased. In addition, intraperitoneal injection of 2-DG significantly decreased IL-1β (0.55 ± 0.15) and IL-6 (0.97 ± 0.20) levels (Figures 6B,D). This suggests that inhibition of glycolysis may inhibit the release of pro-inflammatory factors following inflammatory cell activation.

### 2-DG inhibited the increased glycolysis and pain-related molecules in LPS-stimulated BV2 cells

**Figure 7:**
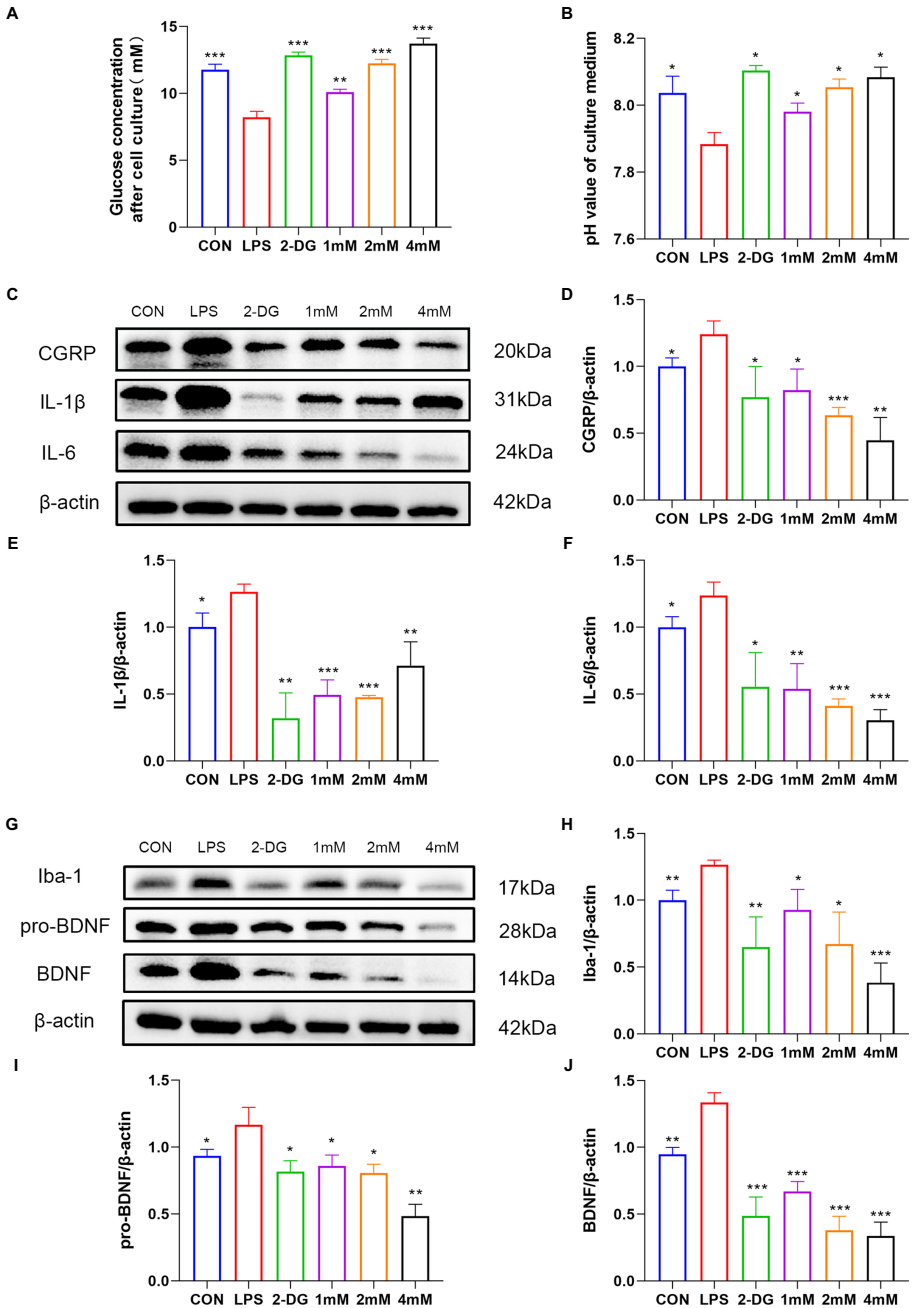
2-DG treatment can inhibit the activation of microglia and the release of pain-related molecules. Residual glucose **(A)** and pH **(B)** of culture supernatants after 12 h stimulation of BV2 by LPS with or without different concentrations of 2-DG. Data were expressed as mean ± SD (*n* = 3 per group). ^***^p < 0.001, ^**^p < 0.01, ^*^p < 0.05. **(C)** Western blot showing the expression of CGRP/IL1-β/IL6 in different groups. **(D)** Quantification of protein levels of(Continued)FIGURE 7 (Continued) CGRP in different groups. Data were expressed as mean ± SD (*n* = 3 per group). ^***^p < 0.001, ^**^p < 0.01, ^*^p < 0.05. **(E)** Quantification of protein levels of IL1-β in different groups. Data were expressed as mean ± SD (*n* = 3 per group). ^***^p < 0.001, ^**^p < 0.01, ^*^p < 0.05. **(F)** Quantification of protein levels of IL6 in different groups. Data were expressed as mean ± SD (*n* = 3 per group). ^***^p < 0.001, ^**^p < 0.01, ^*^p < 0.05. **(G)** Western blot showing the expression of Iba-1/pro-BDNF/BDNF in different groups. **(H)** Quantification of protein levels of Iba-1 in different groups. **(I)** Quantification of protein levels of pro-BDNF in different groups. Data were expressed as mean ± SD (*n* = 3 per group). ^***^p < 0.001, ^**^p < 0.01, ^*^p < 0.05. **(J)** Quantification of protein levels of BDNF in different groups. Data were expressed as mean ± SD (*n* = 3 per group). ^***^p < 0.001, ^**^p < 0.01.

To explore whether there is enhanced glycolysis in activated microglia, we investigated the metabolism of LPS-stimulated BV2 cells. Following microglia activation, the glucose concentration (8.20 ± 0.46) in the medium, which reflects glucose consumption, dropped dramatically (Figure 7A), and the pH (7.88 ± 0.04), which reflects lactate levels, dropped significantly (Figure 7B). Both glucose consumption and changes in pH indicated enhanced glycolysis in activated microglia. Glucose consumption and acidity of the medium were significantly reduced after 2-DG treatment (Figures 7A,B).

To explore the effect of 2-DG on pro-inflammatory cytokine and migraine-related protein expression, IL-6, IL-1β and CGRP production were analyzed by western blotting. Our results showed that the protein levels of IL-6 (1.24 ± 0.15), IL-1β (1.26 ± 0.05) and CGRP (1.24 ± 0.10) were upregulated in activated microglia, whereas 2-DG treatment inhibited it (Figures 7C–F). Taken together, our results suggest that 2-DG has a dose-dependent anti-inflammatory effect and reduces the expression of CGRP (0.82 ± 0.16; 0.63 ± 0.06; 0.45 ± 0.17) in LPS-induced BV2 cells. In order to investigate whether changes in proBDNF/BDNF levels are associated with activation of microglia, we stimulated microglia with endotoxin, a well-recognized activator of microglia. The levels of proBDNF (1.17 ± 0.13) and BDNF (1.33 ± 0.07) secreted by activated microglia were significantly increased, compared with resting microglia. Iba-1 levels (1.26 ± 0.03) were also shown to be higher than the CON group (1.00 ± 0.08). However, the treatment of glycolysis inhibitor 2-DG (0.65 ± 0.23) significantly reduced the expression of Iba-1. This suggests that inhibition of glycolysis inhibits activation of microglia. With the increase of 2-DG concentration, the expression of Iba-1(0.92 ± 0.15; 0.67 ± 0.24; 0.38 ± 0.15) decreased in a dose-dependent manner. In addition, after 2-DG treatment, the expressions of proBDNF (0.85 ± 0.08; 0.80 ± 0.07; 0.48 ± 0.09) and BDNF (0.67 ± 0.07; 0.37 ± 0.11; 0.34 ± 0.10) decreased significantly, also in a dose-dependent manner (Figures 7G–J). This finding proves that inhibition of glycolysis may have a role in migraine through the BDNF pathway.

## Discussion

To determine the role of glycolysis in migraine pathogenesis, we investigated the changes of glycolytic metabolism in the inflammatory soup model and the effect of glycolysis inhibition by 2-DG on inflammatory soup model. The model of IS-induced migraine IS based on the trigeminal vascular theory. When migraine occurs, the injurious stimulus information is transmitted through the trigeminal ganglion and into the brain stem, thalamus, cortex and other pain centers through the sensory nerve upstream conduction system, leading to headache. At the same time, peripheral nerves are activated so that their nerve endings release vasoactive pro-inflammatory peptides, such as CGRP, Substance P, neurokinin A, etc., which in turn trigger vasodilation and migraine symptoms (3). According to previous studies, 2DG with a dose of 500 mg/kg/day has no toxic effect on body weight or general health. The rats were intraperitoneally injected with 2DG at a dose of up to 1,000 mg/kg/day for 14 days, which had no obvious harmful effect on spatial learning and memory (32). However, the dose of 250 mg/kg was related to the reversible decline of exploration behavior, similar to that of rats treated with ketogenic diet (33). Taken together, these results suggest that IS dural infusion stimulation leads to metabolic reprogramming of microglia to increase their glycolytic rates relative to OXPHOS, without much change in key enzymes of the pentose phosphate pathway. 2-DG inhibited hyperalgesia or allodynia and the release of inflammatory factors. This study indicated that inhibition of glycolysis is an effective anti-inflammatory strategy for migraine.

Technological advances in neuroimaging and genetics enable examining various aspects of brain metabolism in migraine patients (34). Magnetic resonance spectroscopy (MRS) enables non-invasive measurements of various substances in brain tissue, particularly some that provide key information about energy metabolism, such as lactate, magnesium, and ATP. The use of 1H-MRS has shown that interictal lactate levels are elevated in migraine patients, speculating that glycolysis in the brains of migraine patients is enhanced during interictal periods, and that longer attack-free periods may normalize subclinical metabolic disturbances (24, 35, 36). After IS stimulation, the genes involved in glycolysis of medulla dorsal horn in migraine model group were significantly higher than those in control group. We also found upregulation of PFKP, a key enzyme of the glycolytic pathway, thus indicating an enhancement of glycolysis in a rat model of migraine. COXIV contributes to ATP synthesis, electron transport and energy metabolism. Dysfunction of COXIV reduces electron flow through the respiratory chain, switches metabolism to glycolysis, induces lactate accumulation and impairs ATP production (37). We found decreased COXIV in the medulla dorsal horn of the IS group. This may suggest the impaired function of mitochondrial in migraine. Furthermore, we explored the expression of G6PD, a key enzyme of the pentose phosphate pathway. However, no significant differences were observed between the CON and IS groups.

The current study demonstrated that the microglial activation and the subsequent release of inflammatory and neuroinflammatory factors play a crucial role in experimental migraine. We showed that migraine is accompanied by the activation of microglia and the release of inflammatory cytokines. Consistent with our research, some studies have found that the transcriptional activity of inflammatory cytokines (IL-6 and TNF-α) and CGRP was increased in trigeminal ganglia, cervical medulla, and pons in NTG-induced migraine model (38). In addition, repeated administration of NTG induced mechanical hyperalgesia and increased NLRP3 and IL-1β expression. Blocking NLRP3 or IL-1β reduced NTG-induced hyperalgesia, and this effect was accompanied by significant inhibition of c-Fos and CGRP levels in TNC (39). We found that microglia in the dorsal horn of medulla were activated and the expression of c-Fos,CGRP, IL-1 and IL-6β increased after IS stimulation. The cells that participate in the pro-inflammatory response, such as activated microglia, must be rapidly energized by glycolysis and high lactate production to trigger inflammation (40). Despite oxygen presence, glucose can be fermented into lactate without entering the tricarboxylic acid cycle, also known as aerobic glycolysis. This aerobic glycolysis is also present in some tumor cells. 2-DG treatment induced apoptosis of glycolytic dependent tumor cells (41). However, aerobic cells with functional mitochondria are able to survive glycolytic inhibition (42). 2-DG mimics sugar deficiency does not make some cells susceptible to death because they maintain OXPHOS function and use alternative carbon sources such as fatty acids and amino acids to synthesize ATP under normoxic conditions (43). In our study, 2-DG treatment inhibited IS-induced astrocyte proliferation in the medulla dorsal horn, but had no significant effect on neuronal loss. Metabolic reprogramming occurs when immune cells are activated to trigger an inflammatory response, despite glycolysis being less efficient than OXPHOS and the immune system prefers glycolysis as a source of energy (23). Microglia in the resting state monitor their environment for any potential injury factors. As soon as exposed to a potentially damaging environment, they are activated and start performing a variety of responses designed to restore homeostasis. To ensure energy requirements, resting microglia predominantly use oxidative phosphorylation and once activated, switch to glycolysis and increase glucose uptake (44, 45). Researchers found that exposure to amyloid-β led to metabolic reprogramming from oxidative phosphorylation to glycolysis in microglia (46). Glycolysis was enhanced in LPS-activated microglia cells, along with the release of inflammatory factors. After the administration of glycolysis inhibitors, with the inhibition of glycolysis, the activation of microglia cells was inhibited and the expression of inflammatory factors was reduced. We concluded that increased glycolysis that accompanies activation of pro-inflammatory cells is also present *in vivo*. Administration of the glycolysis inhibitor 2-DG inhibits microglia activation (Figure 5) and reduces the consequent release of pro-inflammatory cytokines (Figure 6), as well as alleviating migraine-like symptoms.

When glycolysis *in vivo* is inhibited, cells of the body use ketone bodies for energy. Ketogenic diet is a high-fat diet that has been used to control refractory epilepsy by reducing glycolysis within the brain cells. The ketogenic diet also inhibits neuroinflammation in animal models of multiple sclerosis (47, 48). In addition, the ketogenic diet prescription successfully improved short-term symptoms of chronic migraine. It significantly reduced the number of migraine days the patient experienced per month, the number of migraine hours the patient experienced per day, the number of pain medications taken per month, and the intensity of pain (49). Therefore, metabolic modulation may be a promising target for migraine therapy in the future. In our study, we employed preventive treatment. The preventive treatment effectively impeded the development of migraine, manifested by decreased head scratching, increased mechanical pain threshold, and reduced expression of c-Fos and CGRP. CGRP is an essential factor in migraine treatment and diagnosis. The migraine drug triptans inhibit CGRP release, but their vasoconstrictive effects make them unusable in patients with poorly controlled blood pressure (50). In addition, anti-CGRP receptor antibodies (Erenumab) and anti-CGRP antibodies (Galcanezumab and Eptinezumab) have entered the market as prophylactic treatments (51). Our findings suggest that inhibition of glycolysis modulates CGRP and c-Fos expression in the sensory system, highlighting an unexplored role of metabolic regulation on the pathophysiological development of a migraine attack.

Previous studies have suggested that BDNF is a pain-modulator and activated microglia cells promote migraine by synthesizing and releasing BDNF (52). During headache, the incoming information from the meninges releases BDNF through the center to initiate the injurious sensory system of the dura (53). In activated microglia, we observed increased expression of BDNF and its precursor proBDNF. After the administration of glycolysis inhibitor 2-DG, the activation of microglia was inhibited, BDNF and proBDNF were significantly reduced. Glycolytic inhibitors may influence the occurrence of migraine through the Iba-1/proBDNF/BDNF pathway.

Limitations of this study must be admitted. First, considering the effect of estrogen level on the pain behavior of rats (54), only male rats were included in the study. However, migraine is currently believed to be more common in women than men (55), and the role and mechanism of 2-DG in female migraine remains to be further explored. Second, 2-DG May influence migraine-related behaviors through a variety of pathways. 2-DG can inhibit PI3K/Akt/mTOR and up-regulate AMPK pathway to reduce inflammation and oxidative stress (56, 57). This study only discussed the mechanism of glycolysis related to inflammation, while the possible effect of 2-DG on migraine through oxidative stress and PI3K/Akt pathway remains to be explored.

In conclusion, we found that IS dural infusion resulted in enhanced glycolysis and increased production of pro-inflammatory cytokines. The glycolysis inhibitor 2-DG significantly inhibited the symptoms of migraine, and the underlying mechanisms included inhibition of microglia activation, inhibition of Iba-1/proBDNF/BDNF pathway, and the release of inflammatory and neurogenic inflammatory factors. Our current findings link glycolysis to the development of migraine, and further exploration and characterization of these mechanisms will facilitate the role of glycolytic regulation in the treatment of migraine.

## Data availability statement

The original contributions presented in the study are included in the article/supplementary material, further inquiries can be directed to the corresponding author.

## Ethics statement

The animal study was reviewed and approved by Animal Care and Use Committee of Renmin Hospital of Wuhan University.

## Author contributions

TQ: conceptualization, methodology, experiment, validation, and writing—original draft. YaZ: writing—original draft, data curation, and validation. LH: visualization and investigation. ZS: resources and supervision. YuZ: software and validation. YF: writing—review. WH: visualization. LZ: data curation. SF: project administration. ZX: conceptualization, funding acquisition, resources, supervision, and writing—review & editing. All authors contributed to the article and approved the submitted version.

## Funding

National Natural Science Foundation of China (81971055, 82101292, 81471133), Natural Science Foundation of Hubei Province (No. 2020CFB226), and Science Foundation of Buchang Zhiyuan (HIGHER2022094).

## Conflict of interest

The authors declare that the research was conducted in the absence of any commercial or financial relationships that could be construed as a potential conflict of interest.

## Publisher’s note

All claims expressed in this article are solely those of the authors and do not necessarily represent those of their affiliated organizations, or those of the publisher, the editors and the reviewers. Any product that may be evaluated in this article, or claim that may be made by its manufacturer, is not guaranteed or endorsed by the publisher.
